# Flow of Dilute Aqueous Polymer Solutions in a Heterogeneous Porous Medium: Existence Results for Steady and Unsteady Cases

**DOI:** 10.3390/polym18131616

**Published:** 2026-06-29

**Authors:** Evgenii S. Baranovskii, Mikhail A. Artemov

**Affiliations:** Department of Applied Mathematics, Informatics and Mechanics, Voronezh State University, 394018 Voronezh, Russia; artemov_m_a@mail.ru

**Keywords:** non-Newtonian fluids, aqueous polymer solutions, heterogeneous porous media, Navier slip condition, Kelvin–Voigt–Brinkman–Forchheimer equations, weak solutions, global solvability, existence theorem, 76S05, 76D03, 76A10

## Abstract

In this paper, we consider a mathematical model for the flow of a dilute aqueous polymer solution through a heterogeneous porous medium. On the boundary of the flow region, the impermeability condition and a nonlinear Navier-type slip condition are prescribed. Our goal is to investigate the existence of weak solutions to the governing equations of this model, which is a challenging problem for both steady and unsteady flows. To prove the weak solvability, we use a modified Galerkin scheme with special basis elements in appropriate Sobolev spaces. We obtain the existence results without assuming smallness of the model data for the boundary value problem related to steady flows and prove the global-in-time solvability of the initial-boundary value problem describing unsteady flows. Moreover, energy equalities are established for weak solutions possessing additional regularity. Our results can serve as a starting point for further research on the problems under consideration, including numerical analysis and optimal control of polymer fluid flows.

## 1. Introduction

It is widely recognized that the mathematical modeling and analysis of the dynamics of polymer materials are fraught with substantial challenges, many of which remain unresolved [1,2,3,4,5]. Addressing these challenges is not merely a theoretical pursuit but a practical necessity, given the ubiquity of polymer fluids in industrial and biomedical applications.

To date, there is no universal model that can adequately describe flows of all types of polymer fluids. In this paper, we investigate a physically motivated model for the flow of a dilute aqueous polymer solution through a porous medium, given by the following system of nonlinear partial differential equations [6,7,8,9,10]:

(1) $$\begin{array}{c} \rho \left(\partial_{t}\vec{v}+\left(\vec{v}\cdot \nabla \right)\vec{v}\right)-\nabla \cdot \left(\eta_{\mathrm{eff}}\nabla \vec{v}\right)-\nabla \cdot \tau_{p}+\nabla \pi +\beta_{D}\vec{v}+\beta_{F}\rho \left|\vec{v}\right|\vec{v}=\rho \vec{g}, \end{array}$$

(2) $$\begin{array}{c} \mathrm{div}\vec{v}=0. \end{array}$$

Here, we use the following notation:

- *t* denotes time;
- ρ is the fluid density, ρ>0;
- $\vec{v}$ is the flow velocity;
- π is the pressure;
- $\vec{g}$ is the specific body force;
- ∇ stands for the gradient with respect to the spatial variables $x_{1},\ldots ,x_{d}$, where d=2 or 3;
- ∇·τ denotes the vector with the coordinates
  $${\left(\nabla \cdot \;\tau \right)}_{i}:=\sum_{j=1}^{d}\frac{\partial \tau_{ij}}{\partial x_{j}},\;i=1,\ldots ,d;$$
- $\eta_{\mathrm{eff}}$ is the Brinkman (effective viscosity) coefficient, $\eta_{\mathrm{eff}}>0$;
- $\tau_{p}$ is the “polymeric” part of the extra-stress tensor, $\tau_{p}=\alpha \frac{D}{Dt}D\left(\vec{v}\right)$;
- $D(\vec{v})$ is the rate-of-strain tensor, $D\left(\vec{v}\right)=\left(\nabla \vec{v}+{\left(\nabla \vec{v}\right)}^{⊤}\right)/2$;
- α is the relaxation viscosity coefficient, α≥0;
- $\frac{D}{Dt}$ is the material (Stokes) derivative, $\frac{D}{Dt}:=\partial_{t}+\left(\vec{v}\cdot \nabla \right)$;
- $\beta_{D}$ is the Darcy resistance coefficient, $\beta_{D}=2\eta_{f}/K$;
- $\eta_{f}$ is the viscosity of the fluid, $\eta_{f}>0$;
- *K* is the permeability, K>0;
- $\beta_{F}$ is the Forchheimer resistance coefficient, $\beta_{F}\geq 0$.

The Brinkman term $\nabla \cdot (\eta_{\mathrm{eff}}\nabla \vec{v})$ accounts for the momentum diffusion at the macroscale. The Darcy term $\beta_{D}\vec{v}$ characterizes the linear momentum loss due to the porous matrix, whereas the Forchheimer term $\beta_{F}\rho \left|\vec{v}\right|\vec{v}$ introduces a quadratic correction to account for inertial effects at the pore scale. The term $\nabla \cdot \tau_{p}$ in the momentum equation accounts for viscoelastic (relaxation) effects in the polymer solution.

The porous medium is assumed to be isotropic but heterogeneous, meaning that its structural properties vary with position. Consequently, the permeability *K* is a function of the spatial coordinates: $K=K(\vec{x})$. The Darcy and Forchheimer coefficients are likewise spatially dependent: $\beta_{D}=\beta_{D}\left(\vec{x}\right)$ and $\beta_{F}=\beta_{F}\left(\vec{x}\right)$.

The value of the effective viscosity $\eta_{\mathrm{eff}}$ may be either smaller or greater than the fluid viscosity $\eta_{f}$, depending upon a type of porous medium [11,12]. The range of effective viscosity $\eta_{\mathrm{eff}}$ remains a subject of ongoing debate in the literature, with theoretical predictions, numerical simulations, and experimental measurements yielding conflicting results. There is a lack of consensus on this issue. Therefore, many investigations have considered $\eta_{\mathrm{eff}}\equiv \eta_{f}$ (see, e.g., [13,14,15]). Lundgren [16] and Neal and Nader [17] have shown that setting the effective viscosity of the fluid-saturated porous medium equal to the fluid viscosity provides good agreement with experimental data. Taking this into account and noting that the present study focuses on the aqueous polymer solution model for which no reliable information on $\eta_{\mathrm{eff}}$ has been established, we adopt the common and physically reasonable assumption $\eta_{\mathrm{eff}}\equiv \eta_{f}$. In this regard, it is also appropriate to quote Nield [18]: “For the case of media of small or moderate permeability, it probably does not matter much, from a purely empirical as distinct from a scientific viewpoint, whether or not one takes the effective viscosity to be equal to the fluid viscosity, or that divided by the porosity, in the differential equation or the boundary conditions. In that case, the Brinkman term plays a minor role in comparison with the Darcy term.”

Let us introduce the notation $\eta :=2\eta_{f}$ and rewrite Equation (1) as follows:

(3) $$\rho \left(\partial_{t}\vec{v}+\left(\vec{v}\cdot \nabla \right)\vec{v}\right)-\nabla \cdot \tau +\nabla \pi +\beta_{D}\vec{v}+\beta_{F}\rho \left|\vec{v}\right|\vec{v}=\rho \vec{g}$$

with

(4) $$\tau =\eta D\left(\vec{v}\right)+\alpha \frac{D}{Dt}D\left(\vec{v}\right).$$

The adequacy of rheological model (4) with positive values of α has been confirmed by experimental studies. In particular, it has been found to be a suitable constitutive equation for dilute aqueous solutions of polyethylene oxide, polyacrylamide, and guar gum [19,20]. Clearly, in the limiting case where α=0, $\beta_{D}\equiv 0$, and $\beta_{F}\equiv 0$, Equations (3) and (4) simplify to the standard Navier–Stokes system [21,22,23,24,25], which describes dynamics of pure Newtonian fluids in the absence of a porous matrix.

When studying aqueous polymer solutions, the following simplified version of (4) is often used:

(5) $$\tau =\eta D\left(\vec{v}\right)+\alpha \partial_{t}D\left(\vec{v}\right).$$

**Figure 1:**
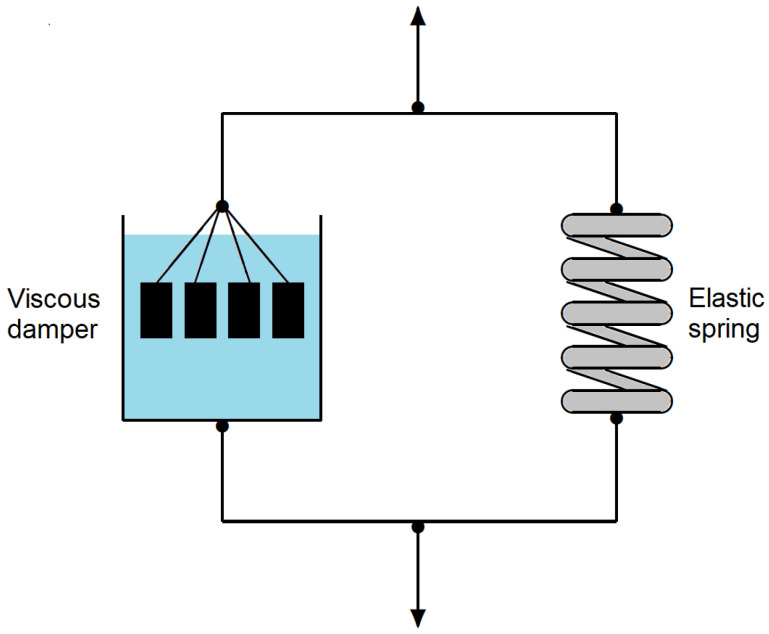
A schematic representation of the viscoelasticity model (5).

This constitutive equation can be justified by considering a Kelvin–Voigt-type mechanical model constructed from a viscous damper and an elastic spring that are arranged in parallel, as shown in Figure 1; see [26] for details.

Combining Equations (3) and (5), we obtain

(6) $$\rho \left(\partial_{t}\vec{v}+\left(\vec{v}\cdot \nabla \right)\vec{v}\right)-\frac{\eta }{2}\Delta \vec{v}-\frac{\alpha }{2}\partial_{t}\Delta \vec{v}+\nabla \pi +\beta_{D}\vec{v}+\beta_{F}\rho \left|\vec{v}\right|\vec{v}=\rho \vec{g},$$

where Δ:=∇·∇ is the Laplace operator.

Equations (6) and (2) are referred to as the *Kelvin–Voigt–Brinkman–Forchheimer equations*. They are of particular relevance in applications involving polymer flow in porous structures and have attracted significant attention in recent years [27,28,29,30].

The well-posedness of the equations of the model for the flow of dilute aqueous polymer solutions with $\beta_{D}\equiv 0$ and $\beta_{F}\equiv 0$ has been studied by many authors. In particular, the seminal works by Oskolkov [31,32,33] should be mentioned. In these papers, sufficient conditions for the solvability of boundary value problems (BVPs) and initial-boundary value problems (IBVPs) for various modifications of the model have been established. The case of a weakly compressible polymer solution was considered by Sviridyuk [34]. Blow-up of solutions of model (6) with a cubic source term has been studied in [35].

The studies mentioned above used zero boundary conditions; that is, the authors considered the regime where fluid particles adhere to the fixed walls of the vessel in which the flow occurs. However, in some problems, the velocity field at boundaries cannot be assumed to be zero. For example, in inflow–outflow problems, as well as boundary control problems for polymer fluid flows, non-homogeneous Dirichlet’s boundary conditions are used [36]. More complex non-homogeneous boundary conditions arise when studying flows with slip effects of solid walls [37,38].

The importance of taking into account boundary slip effects when modeling polymer flows is well known (see, for example, the works [39,40,41,42] and the references therein). It is also known that slippage of particles in a fluid on solid walls can occur in different ways. A detailed discussion of the reasonable slip boundary conditions used in fluid mechanics is given in the review article [43].

Ladyzhenskaya [44] proved the unique solvability of the IBVP in the case of two spatial variables under the so-called *slip condition for particles and vortices*:

$$\vec{n}\times \mathrm{curl}\vec{v}=\vec{0},$$

where $\vec{n}$ denotes the outward unit normal vector to the boundary of a flow domain. In his PhD thesis [45], Kuzmin established the existence of solutions to a problem of optimal control of the flow of the Navier–Stokes–Voigt fluid, taking into account slip effects on solid walls of a vessel. Shi et al. [46] investigated the linear stability of the flow of such fluid in a channel filled with a porous medium subject to asymmetric slip boundary conditions.

In this work, we focus on the following set of boundary conditions:

(7) $$\begin{array}{c} \vec{v}\cdot \vec{n}=0\;\mathrm{on}\;\partial \Omega , \end{array}$$

(8) $$\begin{array}{c} k(\vec{x},|\vec{v}\left|\right)\vec{v}=-{\left(\tau \vec{n}\right)}_{\tan}\;\mathrm{on}\;\partial \Omega , \end{array}$$

where

- Ω is the flow domain, $\Omega \subset R^{d}$ with d=2 or 3;
- ∂Ω denotes the boundary of Ω;
- ${\vec{v}}_{\tan}:=\vec{v}-\left(\vec{v}\cdot \vec{n}\right)\vec{n}$ is the tangential component of the vector $\vec{v}$;
- *k* is the friction coefficient, k:∂Ω×[0,∞)→[0,∞).

Relation (7) represents the impermeability condition, whereas relation (8) is a nonlinear version of Navier’s slip condition [43]. Note that various BVPs and IBVPs for the Stokes and Navier–Stokes systems with Navier-type boundary conditions are considered in many works (see, e.g., [47,48,49,50,51,52]).

The present paper is a continuation of the work [53], in which slip problems related to the model for flow of dilute aqueous polymer solutions are considered in the absence of a porous matrix. Our main aim is to establish the existence of weak solutions for both steady and unsteady flows in a bounded domain without assuming smallness of the model data.

Let us briefly outline the structure of our paper, describe the fluid flow problems considered, and highlight the main results.

In Section 2, we give, for the convenience of the reader, necessary preliminaries and auxiliary results such as a generalized Ascoli–Arzelà theorem (Proposition 1), the Krasnoselskii theorem on the continuity of the superposition operator acting in Lebesgue spaces (Proposition 2), and one result on the solvability of a class of abstract nonlinear equations (Proposition 3).

In Section 3, the stationary version of model (2)–(4) is studied in a bounded domain $\Omega \subset R^{d}$ with d∈{2,3} under boundary conditions (7) and (8). The main result is a theorem on the existence of weak solutions and some of their properties (Theorem 1).

Note that the equations of motion for aqueous polymer solutions include terms containing derivatives of the velocity $\vec{v}$ up to and including third order, while an a priori estimate is available only for the velocity field $\vec{v}$ and its first-order derivatives. Therefore, the standard Galerkin scheme cannot be used to prove the solvability of the corresponding BVP. The technique used in such situations is based on solving a regularized problem with additional terms containing a small numerical parameter, followed by a limiting passage to the original model (see, e.g., [10,32]). However, in the case of nonhomogeneous boundary conditions, the limiting passage is complicated by the appearance (upon integration by parts) of additional terms in the equations, whose magnitudes do not tend to zero. Therefore, to prove Theorem 1, we propose a different approach based on the choice of a special total system of functions in the functional spaces used (see the proof of Proposition 3).

In Section 4, we consider the IBVP for the Kelvin–Voigt–Brinkman–Forchheimer equations under the impermeability condition (7) and the nonlinear slip boundary condition (8). The global-in-time solvability of this IBVP in the weak formulation is established (see Theorem 2). To prove the existence of a weak solution, we use the Faedo–Galerkin method with smooth basis functions. Notably, for this model, it is possible to obtain a priori estimates for the approximate Galerkin solutions that are stronger than those for the Navier–Stokes equations. This is due to the presence of the unsteady term $\partial_{t}\Delta \vec{v}$ in the Kelvin–Voigt–Brinkman–Forchheimer model, which accounts for the relaxation properties of the viscoelastic medium. Based on the obtained estimates and the generalized Ascoli–Arzelà theorem (see Proposition 1), the convergence of the approximate solutions to a weak solution of the original problem is proved, and the continuous dependence of the norm $∥\vec{v}{\left(\cdot ,t\right)∥}_{L^{4}\left(\Omega ,R^{d}\right)}$ of the weak solution with respect to time is established. Moreover, we obtain one regularity criterion and derive the energy equality.

## 2. Preliminaries: Notations, Function Spaces, and Auxiliary Results

### 2.1. Notations and Function Spaces

Let Ω be a bounded Lipschitz domain in $R^{d}$ with d∈{2,3} and Γ:=∂Ω.

Let p∈[1,∞) and ℓ∈N. We will use the Lebesgue space $L^{p}\left(\Omega ,R^{d}\right)$ and the Sobolev space $H^{\ell }\left(\Omega ,R^{d}\right):=W^{\ell ,2}\left(\Omega ,R^{d}\right)$ of functions defined on Ω with values in $R^{d}$. Definitions and an in-depth analysis of properties of these spaces can be found in [54,55,56].

For the sake of brevity, we denote the $L^{2}$-scalar product by parentheses (·,·):

$$\left(\vec{\phi },\vec{\psi }\right):={\left(\vec{\phi },\vec{\psi }\right)}_{L^{2}\left(\Omega ,R^{d}\right)},\;\forall \vec{\phi },\vec{\psi }\in L^{2}\left(\Omega ,R^{d}\right).$$

We will use the boundary trace operator ${\mathrm{Tr}}_{\Gamma }$ (see [55], Chapter III). Recall that for any function $\vec{\phi }\in H^{1}\left(\Omega ,R^{d}\right)$, one can (uniquely) define the boundary trace ${\mathrm{Tr}}_{\Gamma }\;\vec{\phi }\in H^{1/2}\left(\Gamma ,R^{d}\right)$ such that

$${\mathrm{Tr}}_{\Gamma }\;\vec{\phi }=\vec{\phi }{|}_{\Gamma },\;\forall \vec{\phi }\in C^{1}\left(\bar{\Omega },R^{d}\right).$$

Note that the operator ${\mathrm{Tr}}_{\Gamma }:H^{1}\left(\Omega ,R^{d}\right)\to H^{1/2}\left(\Gamma ,R^{d}\right)$ is a continuous linear operator and the following equality holds:

$${\mathrm{Tr}}_{\Gamma }\left(H^{1}\left(\Omega ,R^{d}\right)\right)=H^{1/2}\left(\Gamma ,R^{d}\right).$$

By definition, put

$$\begin{array}{cc} C_{\sigma }^{\infty }\left(\Omega ,R^{d}\right) & :=\left\{\vec{\phi }\in C^{\infty }\left(\bar{\Omega },R^{d}\right):\;\mathrm{div}\;\vec{\phi }=0\;\mathrm{in}\;\Omega \right\},\\ C_{\tan}^{\infty }\left(\Omega ,R^{d}\right) & :=\left\{\vec{\phi }\in C^{\infty }\left(\bar{\Omega },R^{d}\right):\;\vec{\phi }{|}_{\Gamma }\cdot \vec{n}=0\right\},\\ C_{\sigma ,\tan}^{\infty }\left(\Omega ,R^{d}\right) & :=\left\{\vec{\phi }\in C^{\infty }\left(\bar{\Omega },R^{d}\right):\;\mathrm{div}\;\vec{\phi }=0\;\mathrm{in}\;\Omega \;\mathrm{and}\;\vec{\phi }{|}_{\Gamma }\cdot \vec{n}=0\right\},\\ H_{\sigma ,\tan}^{\ell }\left(\Omega ,R^{d}\right) & :=\;\mathrm{the}\;\mathrm{closure}\;\mathrm{of}\;\mathrm{the}\;\mathrm{set}\;C_{\sigma ,\tan}^{\infty }\left(\Omega ,R^{d}\right)\;\mathrm{in}\;\mathrm{the}\;\mathrm{space}\;H^{\ell }\left(\Omega ,R^{d}\right),\\ H_{\sigma ,\tan}^{-1}\left(\Omega ,R^{d}\right) & :=\mathrm{the}\;\mathrm{dual}\mathrm{of}\;\mathrm{the}\;\mathrm{space}\;H_{\sigma ,\tan}^{1}\left(\Omega ,R^{d}\right). \end{array}$$

We will use the following variants of Korn’s inequality (see [57], Chapter 1):

(9) $$\begin{array}{cc} {∥D\left(\vec{\phi }\right)∥}_{L^{2}\left(\Omega ,R^{d\times d}\right)}^{2}+{∥{\mathrm{Tr}}_{\Gamma }\vec{\phi }∥}_{L^{2}\left(\Gamma ,R^{d}\right)}^{2} & \geq \kappa_{1}\left(\Omega \right){∥\vec{\phi }∥}_{H^{1}\left(\Omega ,R^{d}\right)}^{2},\;\forall \vec{\phi }\in H^{1}\left(\Omega ,R^{d}\right), \end{array}$$

(10) $$\begin{array}{cc} {∥D\left(\vec{\phi }\right)∥}_{L^{2}\left(\Omega ,R^{d\times d}\right)}^{2}+{∥\vec{\phi }∥}_{L^{2}\left(\Omega ,R^{d}\right)}^{2} & \geq \kappa_{2}\left(\Omega \right){∥\vec{\phi }∥}_{H^{1}\left(\Omega ,R^{d}\right)}^{2},\;\forall \vec{\phi }\in H^{1}\left(\Omega ,R^{d}\right), \end{array}$$

where $\kappa_{1}\left(\Omega \right)$ and $\kappa_{2}\left(\Omega \right)$ are positive constants.

The symbol ↪ stands for a continuous embedding, while ↪↪ denotes a compact embedding.

For d∈{2,3}, we have

(11) $$H^{1}\left(\Omega ,R^{d}\right)↪L^{p}\left(\Omega ,R^{d}\right),\;1\leq p\leq 6,$$

and

(12) $$H^{1}\left(\Omega ,R^{d}\right)↪↪L^{p}\left(\Omega ,R^{d}\right),\;1\leq p<6.$$

Let *E* be a Banach space and 0<T<∞. By C([0,T];E) we denote the space of all continuous functions from [0,T] into *E* with the max-norm

$${∥v∥}_{C([0,T];E)}:=\max_{t\in [0,T]}{∥v\left(t\right)∥}_{E}.$$

We also will use the space of all continuously differentiable functions:

$$C^{1}\left(\left[0,T\right];E\right):=\left\{v:\left[0,T\right]\to E:\;v\in C\left(\left[0,T\right];E\right)\;\mathrm{and}\;v^{\prime }\in C\left(\left[0,T\right];E\right)\right\}$$

with the norm

$${∥v∥}_{C^{1}\left(\left[0,T\right];E\right)}:={∥v∥}_{C([0,T];E)}+{∥v^{\prime }∥}_{C([0,T];E)}.$$

Here and in the sequel, the prime symbol ′ denotes the derivative with respect to time *t*.

Finally, for given q∈[1,∞), by $L^{q}\left(0,T;E\right)$, we denote the space of $L^{q}$-integrable functions from [0,T] into *E*, which is a Banach space with the norm

$${∥v∥}_{L^{q}\left(0,T;E\right)}:={\left(\int_{0}^{T}{∥v\left(t\right)∥}_{E}^{q}\;dt\right)}^{1/q}.$$

### 2.2. Ascoli–Arzelà Compactness Criterion in Space of Continuous Functions

**Proposition** **1** (generalized Ascoli–Arzelà theorem)**.** *Let E be a Banach space, let M be a subset of the space C([0,T];E), and $M_{t}:=\left\{w\left(t\right):\;w\in M\right\}$ with t∈[0,T]. The set M is relatively compact in the space C([0,T];E) if and only if*

- *the set $M_{t}$ is relatively compact in E, for any t∈(0,T);*
- *for any positive number ϵ, there exists a positive number δ such that the inequality*
$$∥w\left(t_{1}\right)-w\left(t_{2}\right){∥}_{E}\;\leq \;\epsilon$$
*holds for any function w∈M and any numbers $t_{1},t_{2}\in \left[0,T\right]$ such that $|t_{1}-t_{2}|\;\leq \;\delta$.*

A proof of this proposition is given in [58].

### 2.3. Continuity of Superposition Operator in Lebesgue Spaces

**Proposition** **2** (Krasnoselskii’s theorem)**.** *Let U be a bounded domain in the space $R^{n}$. Suppose λ:U×R→R is a function such that*

- *there exist constants $p_{1}\geq 1,$ $p_{2}\geq 1,$ b>0, and a function $a\in L^{p_{2}}\left(U\right)$ such that*
  $$|\lambda \left(\vec{x},y\right)|\leq a\left(\vec{x}\right)+b{\left|y\right|}^{p_{1}/p_{2}},$$
  *for any y∈R and almost every $\vec{x}\in U;$*
- *the function λ(·,y):U→R is measurable for any y∈R;*
- *the function $\lambda (\vec{x},\cdot ):R\to R$ is continuous for almost every $\vec{x}\in U.$*

*Then the superposition operator $N_{\lambda }$ defined by*

$$N_{\lambda }\left[w\right]\left(\cdot \right):=\lambda \left(\cdot ,w\left(\cdot \right)\right),\;\forall w\in L^{p_{2}}\left(U\right),$$

*is continuous and bounded as a mapping from $L^{p_{1}}\left(U\right)$ into $L^{p_{2}}\left(U\right)$.*

A detailed proof of this result can be found in the book [59], Chapter I, § 2.

### 2.4. Sufficient Conditions for Solvability of One Class of Nonlinear Operator Equations

Let *U* and *V* be normed linear spaces. By L(U,V) we denote the space of all continuous linear operators from *U* into *V*.

Let $U^{*}:=L\left(U,R\right)$. The value of a functional $h\in U^{*}$ on an element x∈U is denoted by ${\langle h,x\rangle }_{U^{*}\times U}$.

**Proposition** **3** (Abstract existence result)**.** *Let $E_{1}$ and $E_{2}$ be separable Hilbert spaces such that $E_{2}↪E_{1}$, and let X be a linear (not necessarily closed) subspace of $E_{2}$. Let X be the closure of X in the space $E_{1}$, Y the closure of X in the space $E_{2}$, and Z a Hilbert space such that X↪↪Z.*

Suppose that a linear continuous operator $A:X\to Y^{*}$ and an operator $B:Z\times X\to Y^{*}$ satisfy the following three conditions:

(i)   ${\langle Av,v\rangle }_{Y^{*}\times Y}\geq c_{A}{∥v∥}_{X}^{2}$ with a positive constant $c_{A}$, for any element v∈X;
(ii)  ${\langle B\left(v,v\right),v\rangle }_{Y^{*}\times Y}\geq 0$, for any element v∈X;
(iii) *for any sequences ${\left\{u_{m}\right\}}_{m=1}^{\infty }\subset Z$ and ${\left\{v_{m}\right\}}_{m=1}^{\infty }\subset X$ such that*
(13) $$\begin{array}{cc} u_{m} & \to u_{0}\;strongly\;in\;Z\;as\;m\to \infty , \end{array}$$
(14) $$\begin{array}{cc} v_{m} & \to v_{0}\;weakly\;in\;X\;as\;m\to \infty , \end{array}$$
*the equality*
(15) $$\lim_{m\to \infty }{\langle B\left(u_{m},v_{m}\right),\varphi \rangle }_{Y^{*}\times Y}={\langle B\left(u_{0},v_{0}\right),\varphi \rangle }_{Y^{*}\times Y}$$
*holds for any φ∈X.*

Then, for any functional $f\in Y^{*}$, the operator equation

(16) Av+B(v,v)=f

has at least one solution $v_{*}\in X$.

**Proof.**  Following [53], we consider a sequence ${\left\{\left(x_{i},y_{i}\right)\right\}}_{i=1}^{\infty }$ such that $(x_{i},y_{i})\in X\times Y$ for any i∈N, the set ${\left\{x_{i}\right\}}_{i=1}^{\infty }$ is dense in the space *X*, and the set ${\left\{y_{i}\right\}}_{i=1}^{\infty }$ is dense in the space *Y*.

Since the set X×X is dense in X×Y, we see that there exists a pair $(\varphi_{11},\psi_{11})\in X\times X$ such that

$${∥x_{1}-\varphi_{11}∥}_{X}\leq \frac{1}{2},\;{∥y_{1}-\psi_{11}∥}_{Y}\leq \frac{1}{2}.$$

Moreover, there are pairs $(\varphi_{21},\psi_{21})\in X\times X$ and $(\varphi_{22},\psi_{22})\in X\times X$ such that

$${∥x_{1}-\varphi_{21}∥}_{X}\leq \frac{1}{2^{2}},\;∥y_{1}-\psi_{21}{∥}_{Y}\leq \frac{1}{2^{2}},\;∥x_{2}-\varphi_{22}{∥}_{X}\leq \frac{1}{2^{2}},\;{∥y_{2}-\psi_{22}∥}_{Y}\leq \frac{1}{2^{2}}.$$

Similarly, for each n∈N, there are *n* pairs

$$\left(\varphi_{n1},\psi_{n1}\right),\;\ldots ,\;\left(\varphi_{nn},\psi_{nn}\right)\in X\times X$$

such that

$${∥x_{i}-\varphi_{ni}∥}_{X}\leq \frac{1}{2^{n}},\;{∥y_{i}-\psi_{ni}∥}_{Y}\leq \frac{1}{2^{n}},\;\forall i=1,\ldots ,n.$$

Consider the following sequence

(17) $$\varphi_{11},\;\psi_{11},\;\varphi_{21},\;\psi_{21},\;\varphi_{22},\;\psi_{22},\;\varphi_{31},\;\psi_{31},\ldots$$

It is clear that this sequence is total in the space *X* (that is, the smallest closed subspace containing (17) is the whole space *X*) and is total in *Y*.

Let us perform the following transformations on sequence (17):

1. We eliminate from (17) all elements that can be written as linear combinations of elements with smaller indices.
2. We apply the orthogonalization process (in *X*) to the obtained sequence.

Denote the resulting sequence as ${\left\{\omega_{j}\right\}}_{j=1}^{\infty }$. We obtain an orthonormal basis in *X* such that $\omega_{j}\in X$, for any j∈N, and the sequence ${\left\{\omega_{j}\right\}}_{j=1}^{\infty }$ is total in *Y*.

For an arbitrary given m∈N, we consider the following finite-dimensional problem:

*Find a vector $\vec{\zeta }=\left(\zeta_{1},\ldots ,\zeta_{m}\right)\in R^{m}$ such that*

(18) $$\begin{array}{cc} {\langle Av_{m},\omega_{1}\rangle }_{Y^{*}\times Y}+{\langle B\left(v_{m},v_{m}\right),\omega_{1}\rangle }_{Y^{*}\times Y} & ={\langle f,\omega_{1}\rangle }_{Y^{*}\times Y},\\ {\langle Av_{m},\omega_{2}\rangle }_{Y^{*}\times Y}+{\langle B\left(v_{m},v_{m}\right),\omega_{2}\rangle }_{Y^{*}\times Y} & ={\langle f,\omega_{2}\rangle }_{Y^{*}\times Y},\\ \ldots \ldots \ldots \ldots \ldots \ldots \ldots \ldots \ldots \ldots \ldots \ldots & \ldots \ldots \ldots \ldots \ldots \\ {\langle Av_{m},\omega_{m}\rangle }_{Y^{*}\times Y}+{\langle B\left(v_{m},v_{m}\right),\omega_{m}\rangle }_{Y^{*}\times Y} & ={\langle f,\omega_{m}\rangle }_{Y^{*}\times Y}, \end{array}$$

*where*

$$v_{m}:=\sum_{j=1}^{m}\zeta_{j}\omega_{j}.$$

Let us define a mapping ${\vec{\Phi }}_{m}:R^{m}\to R^{m}$ by the formula

$${\vec{\Phi }}_{m}\left(\zeta \right):=\left(\Phi_{m1}\left(\vec{\zeta }\right),\ldots ,\Phi_{mm}\left(\vec{\zeta }\right)\right),\;\forall \vec{\zeta }\in R^{m},$$

with

$$\Phi_{mj}\left(\vec{\zeta }\right):={\langle Av_{m},\omega_{j}\rangle }_{Y^{*}\times Y}+{\langle B\left(v_{m},v_{m}\right),\omega_{j}\rangle }_{Y^{*}\times Y}$$

and consider a vector $\vec{f}$ given as follows:

$$\vec{f}:=\left({\langle f,\omega_{1}\rangle }_{Y^{*}\times Y},\;\ldots ,\;{\langle f,\omega_{m}\rangle }_{Y^{*}\times Y}\right).$$

Using ${\vec{\Phi }}_{m}$ and $\vec{f}$, we rewrite problem (18) in the concise form

(19) $${\vec{\Phi }}_{m}\left(\vec{\zeta }\right)=\vec{f}.$$

Taking into account conditions (i) and (ii), we obtain

$$\begin{array}{cc} \left({\vec{\Phi }}_{m}\left(\vec{\zeta }\right),\vec{\zeta }\right) & ={\langle Av_{m},v_{m}\rangle }_{Y^{*}\times Y}+{\langle B\left(v_{m},v_{m}\right),v_{m}\rangle }_{Y^{*}\times Y}\\ \; & \geq c_{A}{∥v_{m}∥}_{X}^{2}\\ \; & =c_{A}{∥\vec{\zeta }∥}_{R^{m}}^{2}. \end{array}$$

This means that the mapping ${\vec{\Phi }}_{m}$ is coercive. Moreover, the continuity of A and condition (iii) guarantee that ${\vec{\Phi }}_{m}$ is continuous on $R^{m}$.

Therefore, using Lemma 2.1 from the monograph [60], Chapter III, § 2, we deduce that for any vector $\vec{h}\in R^{m}$, the equation

$${\vec{\Phi }}_{m}\left(\vec{\zeta }\right)=\vec{h}$$

admits a solution; in particular, Equation (19) has a solution. Hence, problem (18) is solvable.

Let $\vec{\zeta }$ be a solution of (18). Multiply the *j*th equation in system (18) by $\zeta_{j}$ and sum the resulting equalities over j=1,…,m. In view of conditions (i) and (ii), we obtain the following inequality

(20) $${∥v_{m}∥}_{X}\leq \frac{1}{c_{A}}{∥f∥}_{Y^{*}},$$

which means that the sequence ${\left\{v_{m}\right\}}_{m=1}^{\infty }$ is bounded in the space *X*. Therefore, there exists an element $v_{*}\in X$ such that

$$v_{m_{s}}\to v_{*}\;\mathrm{weakly}\;\mathrm{in}\;X\;\mathrm{as}\;s\to \infty ,$$

for some subsequence ${\left\{m_{s}\right\}}_{s=1}^{\infty }$. Without loss of generality, we can assume that

(21) $$v_{m}\to v_{*}\;\mathrm{weakly}\;\mathrm{in}\;X\;\mathrm{as}\;m\to \infty .$$

Moreover, since X↪↪Z, we have

(22) $$v_{m}\to v_{*}\;\mathrm{strongly}\;\mathrm{in}\;Z\;\mathrm{as}\;m\to \infty .$$

Let us fix an arbitrary j∈N. Using condition (iii) together with (21) and (22), we can pass to the limit m→∞ in the equality

$${\langle Av_{m},\omega_{j}\rangle }_{Y^{*}\times Y}+{\langle B\left(v_{m},v_{m}\right),\omega_{j}\rangle }_{Y^{*}\times Y}={\langle f,\omega_{j}\rangle }_{Y^{*}\times Y}.$$

As a result, we arrive at

$${\langle Av_{*},\omega_{j}\rangle }_{Y^{*}\times Y}+{\langle B\left(v_{*},v_{*}\right),\omega_{j}\rangle }_{Y^{*}\times Y}={\langle f,\omega_{j}\rangle }_{Y^{*}\times Y}.$$

Since the sequence ${\left\{\omega_{j}\right\}}_{j=1}^{\infty }$ is total in *Y*, we see that the last equality remains valid if we replace $\omega_{j}$ in it with an arbitrary element ω∈Y:

$${\langle Av_{*},\omega \rangle }_{Y^{*}\times Y}+{\langle B\left(v_{*},v_{*}\right),\omega \rangle }_{Y^{*}\times Y}={\langle f,\omega \rangle }_{Y^{*}\times Y}.$$

which implies that $v_{*}$ is a solution of Equation (16). Thus, Proposition 3 is proved. □

## 3. Weak Formulation and Solvability of BVP for Steady-State Flows

Let us consider the system of equations describing the steady-state flow of a polymer solution inside a bounded Lipschitz domain $\Omega \subset R^{d}$, d=2,3, with boundary Γ:

(23) $$\begin{array}{c} \rho \left(\vec{v}\cdot \nabla \right)\vec{v}-\nabla \cdot \left(\eta D\left(\vec{v}\right)+\alpha \left(\vec{v}\cdot \nabla \right)D\left(\vec{v}\right)\right)+\nabla \pi +\beta_{D}\vec{v}+\beta_{F}\rho \left|\vec{v}\right|\vec{v}=\rho \vec{g}\;\mathrm{in}\;\Omega , \end{array}$$

(24) $$\begin{array}{c} \mathrm{div}\vec{v}=0\;\mathrm{in}\;\Omega . \end{array}$$

We supplement this system with the impermeability condition (7) and the following nonlinear slip condition

(25) $$k(\vec{x},|\vec{v}\left|\right)\vec{v}=-{\left(\eta D\left(\vec{v}\right)\vec{n}+\alpha \left(\left(\vec{v}\cdot \nabla \right)D\left(\vec{v}\right)\right)\vec{n}\right)}_{\tan}\;\mathrm{on}\;\Gamma ,$$

which is derived by substituting the extra-stress tensor τ, given by constitutive Equation (4), into relation (8).

To write BVP (23), (24), (7), (25) in dimensionless form, choose a characteristic length *L* and a characteristic speed *V* and define new dimensional constants and functions:

(26) $$\begin{array}{c} {\vec{x}}^{\;⋆}:=\frac{\vec{x}}{L},\;{\vec{v}}^{\;⋆}\left({\vec{x}}^{\;⋆}\right):=\frac{\vec{v}\left(\vec{x}\right)}{V},\;\pi^{⋆}\left({\vec{x}}^{\;⋆}\right):=\frac{\pi (\vec{x})}{\rho V^{2}},\;{\vec{g}}^{\;⋆}\left({\vec{x}}^{\;⋆}\right):=\frac{L}{V^{2}}\vec{g}\left(\vec{x}\right), \end{array}$$

(27) βD⋆:=LβDρV,βF⋆:=LβF,α⋆:=αL2ρ,k⋆(x→⋆,|v→⋆|):=k(x→,|v→|)ρV2,Re:=ρLVη.

Then, expressing the considered BVP in terms of the dimensionless quantities from (26) and (27) and, for brevity, dropping the asterisks, we obtain the following problem:

(28) (v→·∇)v→−∇·1ReD(v→)+α(v→·∇)D(v→)+∇π+βDv→+βF|v→|v→=g→in Ω,divv→=0in Ω,v→·n→=0on Γ,k(x→,|v→|)v→=−1ReD(v→)n→+α((v→·∇)D(v→))n→tanon Γ.

Let $R_{+}:=\left[0,\infty \right)$. Suppose that

(29) $$k\in C(\Gamma \times R_{+},R_{+})$$

and there exist some constants $k_{\min}$ and $k_{\max}$ such that

(30) $$0<k_{\min}\leq k\left(\vec{x},a\right)\leq k_{\max},\;\forall \left(\vec{x},a\right)\in \Gamma \times R_{+}.$$

Moreover, it is assumed that

(31) $$\beta_{D}\in L^{2}\left(\Omega ,R_{+}\right),\;\beta_{F}\in L^{2}\left(\Omega ,R_{+}\right),\;\vec{g}\in L^{2}\left(\Omega ,R^{d}\right).$$

**Definition** **1.** *We shall say that a function $\vec{v}$ is a weak solution of BVP* (28) *if $\vec{v}\in H_{\sigma ,\tan}^{1}\left(\Omega ,R^{d}\right)$ and the following equality holds for any $\vec{w}\in C_{\sigma ,\tan}^{\infty }\left(\Omega ,R^{d}\right)$:*

(32) −∑i=1dviv→,∂w→∂xi+1Re(D(v→),D(w→))−α∑i=1dD(v→),vi∂D(w→)∂xi+∫Γk(x→,|v→|)v→·w→dΓ+(v→,βDw→)+(|v→|v→,βFw→)=(g→,w→).

The above concept of weak solutions match the standard approach for slip problems (compare, for example, with [45,57,61,62]).

Let us explain how relation (32) arises. Assume that a pair $\left(\vec{v},\pi \right)$ is a classical solution of BVP (28) and

(33) τ=1ReD(v→)+α(v→·∇)D(v→).

Then the first equality in (28) can be rewritten as follows:

$$\left(\vec{v}\cdot \nabla \right)\vec{v}-\nabla \cdot \tau +\nabla \pi +\beta_{D}\vec{v}+\beta_{F}\left|\vec{v}\right|\vec{v}=\vec{g}\;\mathrm{in}\;\Omega .$$

Taking the $L^{2}$ scalar product of both parts of the last equality with an arbitrary function $\vec{w}\in C_{\sigma ,\tan}^{\infty }\left(\Omega ,R^{d}\right)$, we have

(34) $$\sum_{i=1}^{d}\left(v_{i}\frac{\partial \vec{v}}{\partial x_{i}},\vec{w}\right)-\left(\nabla \cdot \tau ,\vec{w}\right)+\left(\nabla \pi ,\vec{w}\right)+\left(\vec{v},\beta_{D}\vec{w}\right)+\left(\right|\vec{v}\left|\vec{v},\beta_{F}\vec{w}\right)=\left(\vec{g},\vec{w}\right).$$

Since $\vec{w}$ is solenoidal and tangent to the boundary, the pressure term vanishes: $(\nabla \pi ,\vec{w})=0$. Furthermore, applying the rule of integration by parts to the first and second terms from the left-hand side of (34), we arrive at

(35) $$\begin{array}{cc} \; & -\sum_{i=1}^{d}\left(v_{i}\vec{v},\frac{\partial \vec{w}}{\partial x_{i}}\right)+\left(\tau ,\nabla \vec{w}\right)-\int_{\Gamma }\left(\tau \vec{n}\right)\cdot \vec{w}\;d\Gamma +\left(\vec{v},\beta_{D}\vec{w}\right)+\left(\right|\vec{v}\left|\vec{v},\beta_{F}\vec{w}\right)=\left(\vec{g},\vec{w}\right). \end{array}$$

Using the symmetry property $\tau =\tau^{⊤}$, we derive

(36) $$\begin{array}{cc} \left(\tau ,\nabla \vec{w}\right) & =\frac{1}{2}\left(\tau ,\nabla \vec{w}\right)+\frac{1}{2}\left(\tau^{⊤},{\left(\nabla \vec{w}\right)}^{⊤}\right)\\ \; & =\frac{1}{2}\left(\tau ,\nabla \vec{w}\right)+\frac{1}{2}\left(\tau ,{\left(\nabla \vec{w}\right)}^{⊤}\right)\\ \; & =(\tau ,D\left(\vec{w}\right)). \end{array}$$

Moreover, since $\vec{w}{|}_{\Gamma }\cdot \vec{n}=0$, we have

(37) $$\begin{array}{cc} \int_{\Gamma }\left(\tau \vec{n}\right)\cdot \vec{w}\;d\Gamma & =\int_{\Gamma }\left(\tau \vec{n}\right)\cdot {\vec{w}}_{\tan}\;d\Gamma \\ \; & =\int_{\Gamma }{\left(\tau \vec{n}\right)}_{\tan}\cdot {\vec{w}}_{\tan}\;d\Gamma \\ \; & =\int_{\Gamma }{\left(\tau \vec{n}\right)}_{\tan}\cdot \vec{w}\;d\Gamma . \end{array}$$

Taking into account relations (25), (33), (36), and (37), we derive from (35) equality (32).

Once the velocity field $\vec{v}$ is known, the corresponding pressure π can be determined as an element in the space of distributions in Ω by invoking de Rham’s theorem (see, e.g., the book [22], Chapter I, § 1). The same holds for non-stationary problems. Therefore, in the present paper, we focus on finding the flow velocity.

Note also that by adopting the weak formulation, we relax the regularity requirements for the model data in BVP (28) compared with those of the classical formulation.

The main results of this section are summarized in the following theorem.

**Theorem** **1.** *Suppose that* Ω *is a bounded Lipschitz domain in $R^{d}$ with d∈{2,3}, and α≥0 and conditions* (29)–(31) *are valid; then the following three statements hold.*

(a) *BVP* (28) *has at least one weak solution in the sense of Definition 1.*
(b) If ${\vec{v}}_{*}$ is a weak solution of BVP (28) and ${\vec{v}}_{*}\in H_{\sigma ,\tan}^{3}\left(\Omega ,R^{d}\right)$, then
  (38) 1Re∫Ω|D(v→*)|2dx→+∫Γk(x→,|v→*|)|v→*|2dΓ+∫ΩβD|v→*|2dx→+∫ΩβF|v→*|3dx→=∫Ωg→·v→*dx→.
(c) *The set of weak solutions of BVP* (28) *is sequentially weakly closed in the space $H_{\sigma ,\tan}^{1}\left(\Omega ,R^{d}\right)$.*

**Proof.**  We define the scalar product in the space $H_{\sigma ,\tan}^{1}\left(\Omega ,R^{d}\right)$ as follows:

(39) (ϕ→,ψ→)Hσ,tan1(Ω,Rd):=1Re(D(ϕ→),Dψ→)+kmin∫Γϕ→·ψ→dΓ,∀ϕ→,ψ→∈Hσ,tan1(Ω,Rd).

From Korn’s inequality (9), it follows that the associated Euclidean norm is equivalent to the standard $H^{1}$-norm.

To prove the existence result (a), we apply Proposition 3 with

$$\begin{array}{cc} X & :=C_{\sigma ,\tan}^{\infty }\left(\Omega ,R^{d}\right),\\ E_{1} & :=H^{1}\left(\Omega ,R^{d}\right),\\ E_{2} & :=H^{3}\left(\Omega ,R^{d}\right),\\ X\; & :=\mathrm{the}\;\mathrm{closure}\;\mathrm{of}\;\mathrm{the}\;\mathrm{set}\;C_{\sigma ,\tan}^{\infty }\left(\Omega ,R^{d}\right)\;\mathrm{in}\;\mathrm{the}\;\mathrm{space}\;H^{1}\left(\Omega ,R^{d}\right),\\ Y\; & :=\mathrm{the}\;\mathrm{closure}\;\mathrm{of}\;\mathrm{the}\;\mathrm{set}\;C_{\sigma ,\tan}^{\infty }\left(\Omega ,R^{d}\right)\;\mathrm{in}\;\mathrm{the}\;\mathrm{space}\;H^{3}\left(\Omega ,R^{d}\right),\\ Z\; & :=L^{4}\left(\Omega ,R^{d}\right). \end{array}$$

Let us consider the operators $A:X\to Y^{*}$ and $B:Z\times X\to Y^{*}$, defined by

〈Av→,w→〉Y*×Y:=1Re(D(v→),D(w→))+kmin∫Γv→·w→dΓ

and

$$\begin{array}{cc} {\langle B\left(\vec{u},\vec{v}\right),\vec{w}\rangle }_{Y^{*}\times Y} & :=-\sum_{i=1}^{d}\left(u_{i}\vec{v},\frac{\partial \vec{w}}{\partial x_{i}}\right)-\alpha \sum_{i=1}^{d}\left(D\left(\vec{v}\right),u_{i}\frac{\partial D(\vec{w})}{\partial x_{i}}\right)\\ \; & \;\;\;+\int_{\Gamma }\left(k(\vec{x},|\vec{v}\left|\right)-k_{\min}\right)\vec{v}\cdot \vec{w}\;d\Gamma +\left(\vec{v},\beta_{D}\vec{w}\right)+\left(\right|\vec{v}\left|\vec{v},\beta_{F}\vec{w}\right), \end{array}$$

respectively.

Taking into account the equalities $X=H_{\sigma ,\tan}^{1}\left(\Omega ,R^{d}\right)$ and (39), condition (30), and the two easy-to-verify relations,

$$\begin{array}{cc} \sum_{i=1}^{d}\left(v_{i}\vec{v},\frac{\partial \vec{v}}{\partial x_{i}}\right) & =0,\;\forall \vec{v}\in Y,\\ \sum_{i=1}^{d}\left(D\left(\vec{v}\right),v_{i}\frac{\partial D(\vec{v})}{\partial x_{i}}\right) & =0,\;\forall \vec{v}\in Y, \end{array}$$

we derive

〈Av→,v→〉Y*×Y=1Re(D(v→),D(v→))+kmin∫Γv→·v→dΓ=∥v→∥X2

and

$${\langle B\left(\vec{v},\vec{v}\right),\vec{v}\rangle }_{Y^{*}\times Y}=\int_{\Gamma }\left(k(\vec{x},|\vec{v}\left|\right)-k_{\min}\right)|\vec{v}{|}^{2}\;d\Gamma +\int_{\Omega }\beta_{D}|\vec{v}{|}^{2}\;d\vec{x}+\int_{\Omega }\beta_{F}{\left|\vec{v}\right|}^{3}\;d\vec{x}\geq 0.$$

Using Proposition 2, it is not difficult to show that equality (15) is valid provided that (13) and (14) hold. Thus, conditions (i)–(iii) from Proposition 3 are valid. The application of this proposition yields that Equation (16) has at least one solution $v_{*}\in H_{\sigma ,\tan}^{1}\left(\Omega ,R^{d}\right)$. Clearly, the function $v_{*}$ is a weak solution of BVP (28); that is, statement (a) is proved.

To establish statement (b), we suppose that ${\vec{v}}_{*}\in H_{\sigma ,\tan}^{3}\left(\Omega ,R^{d}\right)$. Clearly, we have

(40) −∑i=1dv*iv→*,∂w→∂xi+1Re(D(v→*),D(w→))−α∑i=1dD(v→*),v*i∂D(w→)∂xi+∫Γk(x→,|v→*|)v→*·w→dΓ+(v→*,βDw→)+(|v→*|v→*,βFw→)=(g→,w→),∀w→∈Cσ,tan∞(Ω,Rd).

Since the set $C_{\sigma ,\tan}^{\infty }\left(\Omega ,R^{d}\right)$ is dense in the space $H_{\sigma ,\tan}^{3}\left(\Omega ,R^{d}\right)$, equality (40) remains valid if we replace $\vec{w}$ with an arbitrary function $\vec{u}$ from the space $H_{\sigma ,\tan}^{3}\left(\Omega ,R^{d}\right)$:

−∑i=1dv*iv→*,∂u→∂xi+1Re(D(v→*),D(u→))−α∑i=1dD(v→*),v*i∂D(u→)∂xi+∫Γk(x→,|v→*|)v→*·u→dΓ+(v→*,βDu→)+(|v→*|v→*,βFu→)=(g→,u→).

Setting $\vec{u}=\vec{v}$ into the last equality and applying integration by parts to the first and third terms in the left-hand side, we arrive at equality (38).

Now we will prove statement (c). Let ${\left\{{\vec{v}}_{j}\right\}}_{j=1}^{\infty }$ be a sequence of weak solutions to BVP (28) such that

(41) $${\vec{v}}_{j}\to \vec{v}\;\mathrm{weakly}\;\mathrm{in}\;\mathrm{the}\;\mathrm{space}\;H_{\sigma ,\tan}^{1}\left(\Omega ,R^{d}\right)\;\mathrm{as}\;j\to \infty .$$

Let us show that $\vec{v}$ is a weak solution to BVP (28). In view of (12), from (41), it follows that

(42) $${\vec{v}}_{j}\to \vec{v}\;\mathrm{strongly}\;\mathrm{in}\;\mathrm{the}\;\mathrm{space}\;L^{4}\left(\Omega ,R^{d}\right)\;\mathrm{as}\;j\to \infty .$$

Taking into account (41) and (42), we obtain

(43) $$\begin{array}{cc} \sum_{i=1}^{d}\left(v_{ji}{\vec{v}}_{j},\frac{\partial \vec{w}}{\partial x_{i}}\right) & \to \sum_{i=1}^{d}\left(v_{i}\vec{v},\frac{\partial \vec{w}}{\partial x_{i}}\right)\;\mathrm{as}\;j\to \infty , \end{array}$$

(44) $$\begin{array}{cc} \sum_{i=1}^{d}\left(D\left({\vec{v}}_{j}\right),v_{ji}\frac{\partial D(\vec{w})}{\partial x_{i}}\right) & \to \sum_{i=1}^{d}\left(D\left(\vec{v}\right),v_{i}\frac{\partial D(\vec{w})}{\partial x_{i}}\right)\;\mathrm{as}\;j\to \infty , \end{array}$$

(45) $$\begin{array}{cc} \left({\vec{v}}_{j},\beta_{D}\vec{w}\right)+\left(\right|{\vec{v}}_{j}\left|{\vec{v}}_{j},\beta_{F}\vec{w}\right) & \to \left(\vec{v},\beta_{D}\vec{w}\right)+\left(\right|\vec{v}\left|\vec{v},\beta_{F}\vec{w}\right)\;\mathrm{as}\;j\to \infty , \end{array}$$

for any function $\vec{w}\in C_{\sigma ,\tan}^{\infty }\left(\Omega ,R^{d}\right)$.

Since the operator ${\mathrm{Tr}}_{\Gamma }:H_{\sigma ,\tan}^{1}\left(\Omega ,R^{d}\right)\to L^{2}\left(\Gamma ,R^{d}\right)$ is compact and (41) holds, we have

(46) $${\vec{v}}_{j}\to \vec{v}\;\mathrm{strongly}\;\mathrm{in}\;\mathrm{the}\;\mathrm{space}L^{2}\left(\Gamma ,R^{d}\right)\;\mathrm{as}\;j\to \infty .$$

Taking into account (30) and (46), by Proposition 2, one can obtain

(47) $$\int_{\Gamma }k(\vec{x},|{\vec{v}}_{j}\left|\right){\vec{v}}_{j}\cdot \vec{w}\;d\Gamma \to \int_{\Gamma }k(\vec{x},|\vec{v}\left|\right)\vec{v}\cdot \vec{w}\;d\Gamma \;\mathrm{as}\;j\to \infty .$$

Let us substitute $\vec{v}={\vec{v}}_{j}$ into (32) and pass to the limit j→∞ in the obtained equality. In view of (41), (43)–(45), and (47), we arrive at equality (32), which is valid for any function $\vec{w}\in C_{\sigma ,\tan}^{\infty }\left(\Omega ,R^{d}\right)$. This means that the function $\vec{v}$ is a weak solution of BVP (28). Thus, the proof of Theorem 1 is complete. □

## 4. Weak Formulation and Global Solvability of IBVP for Unsteady Flows

In this section, we consider evolutionary momentum Equation (6) together with the incompressibility Equation (2) in a space-time cylinder $Q_{T}:=\Omega \times \left(0,T\right)$, where *T* is a fixed positive number (the final time), under impermeability condition (7), the slip boundary condition

(48) $$k(\vec{x},|\vec{v}\left|\right)\vec{v}=-{\left(\eta D\left(\vec{v}\right)\vec{n}+\alpha \partial_{t}D\left(\vec{v}\right)\vec{n}\right)}_{\tan}\;\mathrm{on}\;\Gamma ,$$

and the initial condition

(49) $$\vec{v}{|}_{t=0}={\vec{v}}_{0}\;\mathrm{in}\;\Omega .$$

Note that boundary condition (48) follows from relations (5) and (8).

To write IBVP (6), (2), (7), (48), (49) in dimensionless form, choose a characteristic length *L* and a characteristic speed *V* and introduce the notation

(50) $$\begin{array}{c} {\vec{x}}^{\;⋆}:=\frac{\vec{x}}{L},\;t^{⋆}:=\frac{V}{L}t,\;T^{⋆}:=\frac{V}{L}T,\;{\vec{v}}^{\;⋆}\left({\vec{x}}^{\;⋆},t^{⋆}\right):=\frac{\vec{v}\left(\vec{x},t\right)}{V}, \end{array}$$

(51) $$\begin{array}{c} \pi^{⋆}\left({\vec{x}}^{\;⋆},t^{⋆}\right):=\frac{\pi (\vec{x},t)}{\rho V^{2}},\;{\vec{g}}^{\;⋆}\left({\vec{x}}^{\;⋆},t^{⋆}\right):=\frac{L}{V^{2}}\vec{g}\left(\vec{x},t\right),\;\beta_{D}^{⋆}:=\frac{L\beta_{D}}{\rho V}, \end{array}$$

(52) βF⋆:=LβF,α⋆:=αL2ρ,k⋆(x→⋆,|v→⋆|):=k(x→,|v→|)ρV2,Re:=ρLVη.

Then, expressing the considered IBVP in terms of the dimensionless quantities defined in (50)–(52) and omitting the asterisks for brevity, we obtain the following problem:

(53) ∂tv→+(v→·∇)v→−12ReΔv→−α2∂tΔv→+∇π+βDv→+βF|v→|v→=g→in QT,divv→=0in QT,v→·n→=0on Γ,k(x→,|v→|)v→=−1ReD(v→)n→+α∂tD(v→)n→tanon Γ,v→|t=0=v→0in Ω.

Assume that

(54) $$\begin{array}{c} {\vec{v}}_{0}\in H_{\sigma ,\tan}^{1}\left(\Omega ,R^{d}\right),\;\vec{g}\in C\left(\left[0,T\right];L^{2}\left(\Omega ,R^{d}\right)\right), \end{array}$$

(55) $$\begin{array}{c} \beta_{D}\in L^{2}\left(\Omega ,R_{+}\right),\;\;\;\;\beta_{F}\in L^{2}\left(\Omega ,R_{+}\right). \end{array}$$

**Definition** **2.** *We shall say that a function $\vec{v}$ is a weak solution of IBVP* (53) *if*

-
$\vec{v}\in L^{2}\left(0,T;H_{\sigma ,\tan}^{1}\left(\Omega ,R^{d}\right)\right)\cap C\left(\left[0,T\right];L^{4}\left(\Omega ,R^{d}\right)\right);$

-
$\vec{v}\left(0\right)={\vec{v}}_{0};$

- *for any $\vec{w}\in C_{\sigma ,\tan}^{\infty }\left(\Omega ,R^{d}\right)$, the following equality holds in the distribution sense on (0,T):*
(56) ddt(v→(t),w→)−∑i=1dvi(t)v→(t),∂w→∂xi+1Re(D(v→(t)),D(w→))+αddt(D(v→(t)),D(w→))+∫Γk(x→,|v→(t)|)v→(t)·w→dΓ+(v→(t),βDw→)+(|v→(t)|v→(t),βFw→)=(g→(t),w→).

**Theorem** **2.** *Let* Ω *be a bounded Lipschitz domain in $R^{d}$ with d∈{2,3}. Suppose that α>0, conditions* (29)*,* (30)*,* (54)*, and* (55) *are valid; then the following two statements hold.*

(a) *IBVP* (53) *has at least one weak solution in the sense of Definition 2.*
(b) *If $\vec{v}$ is a weak solution of IBVP* (53) *and $\beta_{F}\in L^{\infty }\left(\Omega \right)$, then*
  (57) $$\vec{v}\in C^{1}\left(\left[0,T\right];H_{\sigma ,\tan}^{1}\left(\Omega ,R^{d}\right)\right)$$
*and the energy equality*
(58) ∫Ω|v→(t)|2dx→+α∫Ω|D(v→(t))|2dx→+2Re∫0t∫Ω|D(v→(s))|2dx→ds+2∫0t∫Γk(x→,|v→(s)|)|v→(s)|2dΓds+2∫0t∫ΩβD|v→(s)|2dx→ds+2∫0t∫ΩβF|v→(s)|3dx→ds=∫Ω|v→0|2dx→+α∫Ω|D(v→0)|2dx→+2∫0t(g→(s),v→(s))ds
*holds for any t∈[0,T].*

**Proof.**  In the framework of the proof of this theorem, we will use the scalar product in the space $H_{\sigma ,\tan}^{1}\left(\Omega ,R^{d}\right)$ defined by the following formula:

$${\left[\vec{\phi },\vec{\psi }\right]}_{H_{\sigma ,\tan}^{1}\left(\Omega ,R^{d}\right)}:=\alpha \left(D\left(\vec{\phi }\right),D\left(\vec{\psi }\right)\right)+\left(\vec{\phi },\vec{\psi }\right),\;\forall \vec{\phi },\vec{\psi }\in H_{\sigma ,\tan}^{1}\left(\Omega ,R^{d}\right);$$

cf. (39). This choice of the scalar product simplifies the derivation of a priori estimates of solutions in the unsteady case.

Note that in view of Korn’s equality (10), the Euclidean norm

$${∥\vec{\phi }∥}_{H_{\sigma ,\tan}^{1}\left(\Omega ,R^{d}\right)}:={\left[\vec{\phi },\vec{\phi }\right]}^{1/2}$$

is equivalent to the standard $H^{1}$-norm. Moreover, we have

(59) $${∥\vec{\phi }∥}_{L^{2}\left(\Omega ,R^{d}\right)}^{2}\leq {∥\vec{\phi }∥}_{H_{\sigma ,\tan}^{1}\left(\Omega ,R^{d}\right)}^{2},\;\forall \vec{\phi }\in H_{\sigma ,\tan}^{1}\left(\Omega ,R^{d}\right),$$

since the norm of $\vec{\phi }$ in $H_{\sigma ,\tan}^{1}\left(\Omega ,R^{d}\right)$ includes the $L^{2}$-norm as a summand.

Let ${\left\{{\vec{w}}_{j}\right\}}_{j=1}^{\infty }$ be an orthonormal basis in $H_{\sigma ,\tan}^{1}\left(\Omega ,R^{d}\right)$ such that

$${\vec{w}}_{j}\in C_{\sigma ,\tan}^{\infty }\left(\Omega ,R^{d}\right),\;\forall j\in N.$$

Let us fix m∈N.

We will construct approximate solutions to IBVP (53) of the form

(60) $${\vec{v}}_{m}\left(t\right):=\sum_{j=1}^{m}q_{mj}\left(t\right){\vec{w}}_{j},$$

where $q_{m1},\ldots ,q_{mm}:\left[0,T\right]\to R$ are unknown functions.

Let us consider an auxiliary problem:

*Find a function ${\vec{q}}_{m}\left(t\right)=\left(q_{m1}\left(t\right),\ldots ,q_{mm}\left(t\right)\right)$ such that*

(61) (v→m′(t),w→1)−∑i=1dvmi(t)v→m(t),∂w→1∂xi+1Re(D(v→m(t)),D(w→1))+α(D(v→m′(t)),D(w→1))+∫Γk(x→,|v→m(t)|)v→m(t)·w→1dΓ+(v→m(t),βDw→1)+(|v→m(t)|v→m(t),βFw→1)=(g→(t),w→1),∀t∈(0,T),…………………………………………………………(v→m′(t),w→m)−∑i=1dvmi(t)v→m(t),∂w→m∂xi+1Re(D(v→m(t)),D(w→m))+α(D(v→m′(t)),D(w→m))+∫Γk(x→,|v→m(t)|)v→m(t)·w→mdΓ+(v→m(t),βDw→m)+(|v→m(t)|v→m(t),βFw→m)=(g→(t),w→m),∀t∈(0,T),

*and*

(62) $${\vec{v}}_{m}\left(0\right)=\sum_{j=1}^{m}{\left({\vec{v}}_{0},{\vec{w}}_{j}\right)}_{H_{\sigma ,\tan}^{1}\left(\Omega ,R^{d}\right)}{\vec{w}}_{j}.$$

Clearly, relations (61) and (62) form the Cauchy problem for a system of nonlinear ordinary differential equations. First of all, we establish a priori estimates for solutions of this problem. Let us assume that a function ${\vec{v}}_{m}$ defined by (60) satisfies (61) and (62). Multiply the *n*th equation in system (61) by $q_{mn}\left(t\right)$ and sum the resulting equalities over n=1,…,m. Taking into account the easy-to-verify relation

$$\sum_{i=1}^{d}\left(v_{mi}\left(t\right){\vec{v}}_{m}\left(t\right),\frac{\partial {\vec{v}}_{m}\left(t\right)}{\partial x_{i}}\right)=0,\;\forall t\in \left(0,T\right),$$

we obtain

(63) (v→m′(t),v→m(t))+1Re(D(v→m(t)),D(v→m(t)))+α(D(v→m′(t)),D(v→m))+∫Γk(x→,|v→m(t)|)|v→m(t)|2dΓ+∫ΩβD|v→m(t)|2dx→+∫ΩβF|v→m(t)|3dx→=(g→(t),v→m(t)),∀t∈(0,T).

Let us rewrite equality (63) as follows:

[v→m′(t),v→m(t)]Hσ,tan1(Ω,Rd)+1Re(D(v→m(t)),D(v→m(t)))+∫Γk(x→,|v→m(t)|)|v→m(t)|2dΓ+∫ΩβD|v→m(t)|2dx→+∫ΩβF|v→m(t)|3dx→=(g→(t),v→m(t)),∀t∈(0,T).

This implies that

(64) $$\frac{d}{dt}{∥{\vec{v}}_{m}\left(t\right)∥}_{H_{\sigma ,\tan}^{1}\left(\Omega ,R^{d}\right)}^{2}\leq 2|\left(\vec{g}\left(t\right),{\vec{v}}_{m}\left(t\right)\right)|,\;\forall t\in \left(0,T\right).$$

Using (59) and Young’s inequality, we derive from (64) the chain of estimates:

(65) $$\begin{array}{cc} \frac{d}{ds}{∥{\vec{v}}_{m}\left(s\right)∥}_{H_{\sigma ,\tan}^{1}\left(\Omega ,R^{d}\right)}^{2} & \leq 2|(\vec{g}\left(s\right),{\vec{v}}_{m}\left(s\right))|\\ \; & \leq \;∥\vec{g}{\left(s\right)∥}_{L^{2}\left(\Omega ,R^{d}\right)}^{2}+{∥{\vec{v}}_{m}\left(s\right)∥}_{L^{2}\left(\Omega ,R^{d}\right)}^{2}\\ \; & \leq \;∥\vec{g}{\left(s\right)∥}_{L^{2}\left(\Omega ,R^{d}\right)}^{2}+{∥{\vec{v}}_{m}\left(s\right)∥}_{H_{\sigma ,\tan}^{1}\left(\Omega ,R^{d}\right)}^{2}\;,\;\forall s\in \left(0,T\right). \end{array}$$

Integrating (65) from 0 to *t* and applying Grönwall’s inequality, we obtain

(66) $$\begin{array}{cc} {∥{\vec{v}}_{m}\left(t\right)∥}_{H_{\sigma ,\tan}^{1}\left(\Omega ,R^{d}\right)}^{2} & \leq \exp\left(T\right){∥{\vec{v}}_{0}∥}_{H_{\sigma ,\tan}^{1}\left(\Omega ,R^{d}\right)}^{2}\\ \; & \;+\exp\left(T\right)\int_{0}^{T}{∥\vec{g}\left(s\right)∥}_{L^{2}\left(\Omega ,R^{d}\right)}^{2}\;ds,\;\forall t\in \left(0,T\right). \end{array}$$

From (60) and (66), it follows that

(67) $$\max_{t\in [0,T]}\sum_{j=1}^{m}q_{mj}^{2}\left(t\right)\leq \exp\left(T\right)∥{\vec{v}}_{0}{∥}_{H_{\sigma ,\tan}^{1}\left(\Omega ,R^{d}\right)}^{2}+\exp\left(T\right)\int_{0}^{T}{∥\vec{g}\left(s\right)∥}_{L^{2}\left(\Omega ,R^{d}\right)}^{2}\;ds.$$

This estimate makes it possible to deduce that the Cauchy problem related to (61) and (62) is solvable on the whole segment [0,T].

Now we will establish an estimate, independent of the parameter *m*, for the time derivative of the function ${\vec{v}}_{m}$.

Let us multiply the *n*th equation in system (61) by $q_{mn}^{\prime }\left(t\right)$ and sum the resulting equalities over n=1,…,m. This gives

(68) v→m′(t),v→m′(t)−∑i=1dvmi(t)v→m(t),∂v→m′(t)∂xi+1Re(D(v→m(t)),D(v→m′(t)))+α(D(v→m′(t)),D(v→m′(t)))+∫Γk(x→,|v→m(t)|)v→m(t)·v→m′(t)dΓ+∫ΩβDv→m(t)·v→m′(t)dx→+∫ΩβF|v→m(t)|v→m(t)·v→m′(t)dx→=(g→(t),v→m′(t)),∀t∈(0,T).

Clearly, the second and seventh terms on the left-hand side of (68) are the most delicate to estimate. Using Hölder’s inequality with exponents 4,4,2 (since 1/4+1/4+1/2=1) and embedding (11) with p=4, we obtain

$$\begin{array}{cc} \left|\sum_{i=1}^{d}\left(v_{mi}\left(t\right){\vec{v}}_{m}\left(t\right),\frac{\partial {\vec{v}}_{m}^{\;\prime }\left(t\right)}{\partial x_{i}}\right)\right|\; & \leq c_{1}∥{\vec{v}}_{m}{\left(t\right)∥}_{L^{4}\left(\Omega ,R^{d}\right)}^{2}\;{∥\nabla {\vec{v}}_{m}^{\;\prime }\left(t\right)∥}_{L^{2}\left(\Omega ,R^{d\times d}\right)}\\ \; & \leq c_{2}∥{\vec{v}}_{m}{\left(t\right)∥}_{H_{\sigma ,\tan}^{1}\left(\Omega ,R^{d}\right)}^{2}\;{∥\nabla {\vec{v}}_{m}^{\;\prime }\left(t\right)∥}_{L^{2}\left(\Omega ,R^{d\times d}\right)}. \end{array}$$

Here and below, the symbol *c* with a subscript denotes a constant that depends only on the model parameters and is independent of *m*.

From (66), it follows that the norm ${∥{\vec{v}}_{m}\left(t\right)∥}_{H_{\sigma ,\tan}^{1}\left(\Omega ,R^{d}\right)}$ is uniformly bounded in *m* and *t*; hence, for any ε>0,

$$\left|\sum_{i=1}^{d}\left(v_{mi}\left(t\right){\vec{v}}_{m}\left(t\right),\frac{\partial {\vec{v}}_{m}^{\;\prime }\left(t\right)}{\partial x_{i}}\right)\right|\leq \varepsilon {∥{\vec{v}}_{m}^{\;\prime }\left(t\right)∥}_{H_{\sigma ,\tan}^{1}\left(\Omega ,R^{d}\right)}^{2}+c_{\varepsilon }.$$

The term containing $\beta_{F}$ is estimated using Hölder’s inequality with exponents 2,6,6,6 (since 1/2+1/6+1/6+1/6=1) as follows:

$$\left|\int_{\Omega }\beta_{F}\left|{\vec{v}}_{m}\left(t\right)\right|{\vec{v}}_{m}\left(t\right)\cdot {\vec{v}}_{m}^{\;\prime }\left(t\right)\;d\vec{x}\right|\leq ∥\beta_{F}{∥}_{L^{2}\left(\Omega ,R\right)}∥{\vec{v}}_{m}{\left(t\right)∥}_{L^{6}\left(\Omega ,R^{d}\right)}^{2}\;{∥{\vec{v}}_{m}^{\;\prime }\left(t\right)∥}_{L^{6}\left(\Omega ,R^{d}\right)}.$$

By embedding (11) with p=6 and the uniform bound from (66), we derive

$$\begin{array}{cc} {∥{\vec{v}}_{m}\left(t\right)∥}_{L^{6}\left(\Omega ,R^{d}\right)} & \leq c_{3}{∥{\vec{v}}_{m}\left(t\right)∥}_{H_{\sigma ,\tan}^{1}\left(\Omega ,R^{d}\right)}\leq c_{4},\\ {∥{\vec{v}}_{m}^{\;\prime }\left(t\right)∥}_{L^{6}\left(\Omega ,R^{d}\right)} & \leq c_{5}{∥{\vec{v}}_{m}^{\;\prime }\left(t\right)∥}_{H_{\sigma ,\tan}^{1}\left(\Omega ,R^{d}\right)}. \end{array}$$

Thus, for any ε>0, we have

$$\left|\int_{\Omega }\beta_{F}\left|{\vec{v}}_{m}\left(t\right)\right|{\vec{v}}_{m}\left(t\right)\cdot {\vec{v}}_{m}^{\;\prime }\left(t\right)\;d\vec{x}\right|\leq \varepsilon {∥{\vec{v}}_{m}^{\;\prime }\left(t\right)∥}_{H_{\sigma ,\tan}^{1}\left(\Omega ,R^{d}\right)}^{2}+c_{\varepsilon }.$$

The remaining terms in (68) are treated similarly using the Cauchy–Schwarz and Young inequalities, together with (30), (55), and the uniform bounds from (66). Collecting all estimates and choosing an ε that is sufficiently small, we obtain

(69) $$\max_{t\in [0,T]}{∥{\vec{v}}_{m}^{\;\prime }\left(t\right)∥}_{H_{\sigma ,\tan}^{1}\left(\Omega ,R^{d}\right)}\leq c_{6}.$$

From estimates (66) and (69), it follows that the set ${\left\{{\vec{v}}_{m}\right\}}_{m=1}^{\infty }$ is bounded in the space $C^{1}\left(\left[0,T\right];H_{\sigma ,\tan}^{1}\left(\Omega ,R^{d}\right)\right)$.

Since $H_{\sigma ,\tan}^{1}\left(\Omega ,R^{d}\right)$ is compactly embedded in $L^{4}\left(\Omega ,R^{d}\right)$, by Proposition 1, we deduce

$$C^{1}\left(\left[0,T\right];H_{\sigma ,\tan}^{1}\left(\Omega ,R^{d}\right)\right)↪↪C\left(\left[0,T\right];L^{4}\left(\Omega ,R^{d}\right)\right).$$

Therefore, the set ${\left\{{\vec{v}}_{m}\right\}}_{m=1}^{\infty }$ is relatively compact in the space $C(\left[0,T\right];L^{4}\left(\Omega ,R^{d}\right))$, and there exists a function ${\vec{v}}_{*}\in C\left(\left[0,T\right];L^{4}\left(\Omega ,R^{d}\right)\right)$ such that

$${\vec{v}}_{m_{j}}\to {\vec{v}}_{*}\;\mathrm{strongly}\;\mathrm{in}\;C\left(\left[0,T\right];L^{4}\left(\Omega ,R^{d}\right)\right)\;\mathrm{as}\;j\to \infty ,$$

for some subsequence $m_{j}$. Without loss of generality, it can be assumed that

(70) $${\vec{v}}_{m}\to {\vec{v}}_{*}\;\mathrm{strongly}\;\mathrm{in}\;C\left(\left[0,T\right];L^{4}\left(\Omega ,R^{d}\right)\right)\;\mathrm{as}\;m\to \infty ,$$

and moreover, in view of estimate (66), we have

(71) $${\vec{v}}_{m}\to {\vec{v}}_{*}\;\mathrm{weakly}\;\mathrm{in}\;L^{2}\left(0,T;H_{\sigma ,\tan}^{1}\left(\Omega ,R^{d}\right)\right)\;\mathrm{as}\;m\to \infty .$$

Let *n* be an arbitrary positive integer, m≥n and ξ:R→R a smooth function such that

supp(ξ)⊂(0,T).

We take the scalar product in $L^{2}\left(0,T\right)$ of both sides of the *n*th equality in system (61) with the function ξ to obtain

(72) ∫0T(v→m′(t),w→n)ξ(t)dt−∫0T∑i=1dvmi(t)v→m(t),∂w→n∂xiξ(t)dt+1Re∫0T(D(v→m(t)),D(w→n))ξ(t)dt+∫0Tα(D(v→m′(t)),D(w→n))ξ(t)dt+∫0T∫Γk(x→,|v→m(t)|)v→m(t)·w→ndΓξ(t)dt+∫0T(v→m(t),βDw→n)ξ(t)dt+∫0T(|v→m(t)|v→m,βFw→n)ξ(t)dt=∫0T(g→(t),w→n)ξ(t)dt.

Let us apply the rule of integration by parts the first and fourth terms from the left-hand side of (72) and pass to the limit as m→∞. Taking into account (70) and (71), we arrive at

(73) −∫0T(v→*(t),w→n)ξ′(t)dt−∫0T∑i=1dv*i(t)v→*(t),∂w→n∂xiξ(t)dt+1Re∫0T(D(v→*(t)),D(w→n))ξ(t)dt−∫0Tα(D(v→*(t)),D(w→n))ξ′(t)dt+∫0T∫Γk(x→,|v→*(t)|)v→*(t)·w→ndΓξ(t)dt+∫0T(v→*(t),βDw→n)ξ(t)dt+∫0T(|v→*(t)|v→*,βFw→n)ξ(t)dt=∫0T(g→(t),w→n)ξ(t)dt.

Since ${\left\{{\vec{w}}_{j}\right\}}_{j=1}^{\infty }$ is an orthonormal basis in $H_{\sigma ,\tan}^{1}\left(\Omega ,R^{d}\right)$, equality (73) remains valid if we replace ${\vec{w}}_{n}$ in it with an arbitrary function $\vec{w}\in C_{\sigma ,\tan}^{\infty }\left(\Omega ,R^{d}\right)$. Thus, the function ${\vec{v}}_{*}$ is a weak solution of IBVP (53).

Further, we will prove statement (b).

Since $H_{\sigma ,\tan}^{1}\left(\Omega ,R^{d}\right)$ is a Hilbert space, the Riesz representation theorem guarantees the existence of the isometric isomorphism R from $H_{\sigma ,\tan}^{1}\left(\Omega ,R^{d}\right)$ onto its dual $H_{\sigma ,\tan}^{-1}\left(\Omega ,R^{d}\right)$, defined by

$${\langle R\vec{u},\vec{\varphi }\rangle }_{H_{\sigma ,\tan}^{-1}\left(\Omega ,R^{d}\right)\times H_{\sigma ,\tan}^{1}\left(\Omega ,R^{d}\right)}\;:={\left(\vec{u},\vec{\varphi }\right)}_{H_{\sigma ,\tan}^{1}\left(\Omega ,R^{d}\right)},\;\forall \vec{u},\vec{\varphi }\in H_{\sigma ,\tan}^{1}\left(\Omega ,R^{d}\right).$$

Clearly, we have

(74) $$R\vec{v}\in L^{2}\left(0,T;H_{\sigma ,\tan}^{-1}\left(\Omega ,R^{d}\right)\right).$$

Moreover, from equality (56) and $\beta_{F}\in L^{\infty }\left(\Omega \right)$, it follows that

(75) $${\left(R\vec{v}\right)}^{\prime }\in L^{2}\left(0,T;H_{\sigma ,\tan}^{-1}\left(\Omega ,R^{d}\right)\right),$$

which, together with inclusion (74), yield that

(76) $$R\vec{v}\in C\left(\left[0,T\right];H_{\sigma ,\tan}^{-1}\left(\Omega ,R^{d}\right)\right),$$

and hence,

(77) $$\vec{v}=R^{-1}\left(R\vec{v}\right)\in C\left(\left[0,T\right];H_{\sigma ,\tan}^{1}\left(\Omega ,R^{d}\right)\right).$$

From (56) and (77), it follows that

(78) $${\left(R\vec{v}\right)}^{\prime }\in C\left(\left[0,T\right];H_{\sigma ,\tan}^{-1}\left(\Omega ,R^{d}\right)\right).$$

In view of (76) and (78), the following inclusion holds:

(79) $$R\vec{v}\in C^{1}\left(\left[0,T\right];H_{\sigma ,\tan}^{-1}\left(\Omega ,R^{d}\right)\right),$$

and hence,

$$\vec{v}=R^{-1}\left(R\vec{v}\right)\in C^{1}\left(\left[0,T\right];H_{\sigma ,\tan}^{1}\left(\Omega ,R^{d}\right)\right);$$

that is, we have proved that inclusion (57) is true.

Let us rewrite (56) as follows:

(80) (v→′(t),w→)−∑i=1dvi(t)v→(t),∂w→∂xi+1Re(D(v→(t)),D(w→))+α(D(v→′(t)),D(w→))+∫Γk(x→,|v→(t)|)v→(t)·w→dΓ+(v→(t),βDw→)+(|v→(t)|v→(t),βFw→)=(g→(t),w→),∀w→∈Hσ,tan1(Ω,Rd) and t∈[0,T].

Setting $\vec{w}=\vec{v}$ into (80), we obtain

(v→′(t),v→(t))−∑i=1dvi(t)v→(t),∂v→(t)∂xi+1Re(D(v→(t)),D(v→(t)))+α(D(v→′(t)),D(v→(t)))+∫Γk(x→,|v→(t)|)v→(t)·v→(t)dΓ+(v→(t),βDv→(t))+(|v→(t)|v→(t),βFv→(t))=(g→(t),v→(t))∀t∈[0,T],

where

(81) 12dds∫Ω|v→(s)|2dx→+α∫Ω|D(v→(s))|2dx→+1Re∫Ω|D(v→(s))|2dx→+∫Γk(x→,|v→(s)|)|v→(s)|2dΓ+∫ΩβD|v→(s)|2dx→+∫ΩβF|v→(s)|3dx→=(g→(s),v→(s)),∀s∈[0,T].

Integrating (81) from 0 to *t*, we obtain

(82) 12∫Ω|v→(t)|2dx→+α∫Ω|D(v→(t))|2dx→−∫Ω|v→(0)|2dx→−α∫Ω|D(v→(0))|2dx→+1Re∫0t∫Ω|D(v→(s))|2dx→ds+∫0t∫Γk(x→,|v→(s)|)|v→(s)|2dΓds+∫0t∫ΩβD|v→(s)|2dx→ds+∫0t∫ΩβF|v→(s)|3dx→ds=∫0t(g→(s),v→(s))ds,∀t∈[0,T].

Furthermore, taking into account the initial condition $\vec{v}\left(0\right)={\vec{v}}_{0}$, we derive from (82) energy equality (58). Thus, Theorem 2 is completely proved. □

## 5. Conclusions

This paper provides a rigorous existence analysis for the model of a dilute aqueous polymer solution flowing through a porous medium with a nonlinear Navier-type slip condition on the flow region boundary. The main mathematical difficulty in constructing solutions lies in the degeneracy of the equations of the model and the inhomogeneity of the boundary conditions. This difficulty has been overcome by a modified Galerkin method using carefully tailored basis functions in Sobolev spaces and by the derivation of suitable a priori estimates.

Two key results are worth highlighting. First, we have proven the existence of weak solutions for steady flows without any smallness assumption on the data—an advance over earlier results that relied on the vanishing viscosity method, which works only for homogeneous boundary conditions. Second, we have established the global-in-time solvability for unsteady flows, showing that solutions are bounded and continuous in time under natural assumptions on the model data. Additionally, for solutions possessing extra regularity, we have derived energy equalities, which are essential for subsequent analysis of the dynamics of polymer flows.

From a broader perspective, our results fill a gap in the scientific literature on non-Newtonian flows in porous media, where most existing studies assume either the no-slip boundary condition [10,27] or a slip law with a constant friction coefficient [46,63]. The nonlinear slip condition considered here is more general and realistic for practical applications.

Despite the above advances, several important questions remain open. Uniqueness of weak solutions, for instance, is not guaranteed by our approach and would require additional assumptions on the model data as well as more refined estimates. Furthermore, the existence of strong and classical solutions, together with the regularity of weak solutions, calls for further investigation. Nevertheless, our existence theory already justifies the use of the Galerkin method for obtaining a correct numerical solution, and the energy equalities furnish natural tools for error analysis.

Finally, the proposed approach can be extended to the analysis of more complex polymer fluids (e.g., some Rivlin–Ericksen fluids [64,65]) under more general slip conditions, such as those involving thresholds or temperature-dependent friction. Thus, this paper not only resolves specific existence problems but also provides a foundation for future analytical and numerical studies of polymer solution flows in porous media with boundary slip effects. That is particularly important for practical problems concerning filtration, enhanced oil recovery, biomedical porous structures, or polymer processing.

## Data Availability

The original contributions presented in this study are included in the article. Further inquiries can be directed to the corresponding author.
